# Identifying trial recruitment uncertainties using a James Lind Alliance Priority Setting Partnership – the PRioRiTy (Prioritising Recruitment in Randomised Trials) study

**DOI:** 10.1186/s13063-018-2544-4

**Published:** 2018-03-01

**Authors:** Patricia Healy, Sandra Galvin, Paula R. Williamson, Shaun Treweek, Caroline Whiting, Beccy Maeso, Christopher Bray, Peter Brocklehurst, Mary Clarke Moloney, Abdel Douiri, Carrol Gamble, Heidi R. Gardner, Derick Mitchell, Derek Stewart, Joan Jordan, Martin O’Donnell, Mike Clarke, Sue H. Pavitt, Eleanor Woodford Guegan, Amanda Blatch-Jones, Valerie Smith, Hannah Reay, Declan Devane

**Affiliations:** 1 Health Research Board – Trials Methodology Research Network, Galway, Ireland; 2 School of Nursing and Midwifery, NUI Galway, Galway, Ireland; 3 MRC North West Hub for Trials Methodology, University of Liverpool, Liverpool, UK; 4 Health Services Research Unit, University of Aberdeen, Aberdeen, UK; 5 James Lind Alliance, Wessex Institute, University of Southampton, Southampton, UK; 6 Oxford University Hospitals NHS Foundation Trust, Oxford, UK; 7 Birmingham Clinical Trials Unit, University of Birmingham, Birmingham, UK; 8 Health Research Institute, University of Limerick, Limerick, Ireland; 9 Division of Health and Social Care Research, King’s College London, London, UK; 10 Irish Platform for Patient Organisations, Science and Industry (IPPOSI), Dublin, Ireland; 11 National Institute for Health Research, London, England, UK; 12 HRB-Clinical Research Facility Galway, NUI Galway, Galway, Ireland; 13 Centre for Public Health, Queen’s University Belfast, Belfast, UK; 14 Dental Translational and Clinical Research Unit (DenTCRU), Leeds NIHR CRF, University of Leeds, Leeds, UK; 15 NIHR Evaluation Trials and Studies Coordinating Centre (NETSCC), University of Southampton, Southampton, UK; 16 School of Nursing & Midwifery, Trinity College Dublin, Dublin, Ireland; 17 National Institute for Health Research-Clinical Research Network (NIHR CRN), Birmingham, England, UK

**Keywords:** Recruitment challenges, Participation in randomised trials, Survey, Priority setting partnership, James Lind Alliance, Trial methodology

## Abstract

**Background:**

Despite the problem of inadequate recruitment to randomised trials, there is little evidence to guide researchers on decisions about how people are effectively recruited to take part in trials. The PRioRiTy study aimed to identify and prioritise important unanswered trial recruitment questions for research. The PRioRiTy study - Priority Setting Partnership (PSP) included members of the public approached to take part in a randomised trial or who have represented participants on randomised trial steering committees, health professionals and research staff with experience of recruiting to randomised trials, people who have designed, conducted, analysed or reported on randomised trials and people with experience of randomised trials methodology.

**Methods:**

This partnership was aided by the James Lind Alliance and involved eight stages: (i) identifying a unique, relevant prioritisation area within trial methodology; (ii) establishing a steering group (iii) identifying and engaging with partners and stakeholders; (iv) formulating an initial list of uncertainties; (v) collating the uncertainties into research questions; (vi) confirming that the questions for research are a current recruitment challenge; (vii) shortlisting questions and (viii) final prioritisation through a face-to-face workshop.

**Results:**

A total of 790 survey respondents yielded 1693 open-text answers to 6 questions, from which 1880 potential questions for research were identified. After merging duplicates, the number of questions was reduced to 496. Questions were combined further, and those that were submitted by fewer than 15 people and/or fewer than 6 of the 7 stakeholder groups were excluded from the next round of prioritisation resulting in 31 unique questions for research. All 31 questions were confirmed as being unanswered after checking relevant, up-to-date research evidence. The 10 highest priority questions were ranked at a face-to-face workshop. The number 1 ranked question was “How can randomised trials become part of routine care and best utilise current clinical care pathways?” The top 10 research questions can be viewed at www.priorityresearch.ie.

**Conclusion:**

The prioritised questions call for a collective focus on normalising trials as part of clinical care, enhancing communication, addressing barriers, enablers and motivators around participation and exploring greater public involvement in the research process.

## Background

Challenges in how randomised trials are designed and conducted are commonly experienced at various stages of the trial process. Uncertainties remain on many aspects of the trial process, from planning and design to conduct, analysis, reporting and dissemination.

Participant recruitment is critical to the success of every randomised trial yet optimising recruitment remains a difficult, ongoing challenge for the trial community. Evidence suggests that less than 50% of trials meet their recruitment target with or without an extension [1, 2]. Difficulties in recruitment often result in delays and additional costs in conducting trials and additional costs associated with the need for extensions. Having to commit additional resources for recruitment efforts may also impact negatively on the quality of follow up for those already recruited, further compromising the trial outcome. Difficulties arise in using the trial results to make informed decisions about clinical care if they have failed to reach the recruitment target necessary for an adequately powered study. From a funder perspective, inadequate recruitment wastes available funding and the question the trial was funded to answer remains unanswered, leaving treatment decisions uncertain. Furthermore, a number of systematic reviews, focusing on interventions to improve recruitment, reveal a shortage of high-quality evidence from randomised evaluations of trial recruitment interventions, and what little evidence that is available has a very narrow scope [2–5]. This is reinforced by Tudur-Smith et al. (2014) who conducted a Delphi study with Clinical Trials Units (CTUs) in the United Kingdom (UK) to identify topics of importance and to establish consensus for research priorities around trial methodology with “Methods to boost recruitment in trials” being identified as the highest priority [6].

For these reasons, it is important that research into how trial recruitment might be improved is conducted urgently so that scarce resources might be directed to areas considered important by key stakeholders. Research questions that are both important to stakeholders and have not been answered to date, may be identified and prioritised through a priority setting partnership (PSP) [7]. This paper reports on the processes and prioritised questions for research identified by the Prioritising Recruitment in Randomised Trials Priority Setting Partnership (PRioRiTy PSP). PSPs have previously used the James Lind Alliance (JLA) method to bring relevant stakeholders together to jointly identify priorities for research concerning treatment for conditions and illnesses [8, 9].

The role of the PSP is to identify questions for research (or “uncertainties”) that are both important to stakeholders and have not been answered to date, and to then prioritise these through engagement across the various stakeholder groups [7]. Prioritised questions are usually broad over-arching questions, for which several more specific questions might be identified. The PRioRiTy PSP was a collaborative project by the Health Research Board Trials Methodology Research Network (HRB-TMRN; https://www.hrb-tmrn.ie) in Ireland with the support of the JLA in the UK. The HRB-TMRN is an all-Ireland collaborative network, which seeks to improve the planning, design, conduct, analysis, reporting and dissemination of randomised trials nationally. The HRB-TMRN was established in 2015 and endeavours to improve the understanding of trial methodology nationally through a suite of activities including training and education, online support and primary methodology research. The JLA is a non-profit making initiative that was established in 2004. The JLA is funded by the National Institute for Health Research (NIHR) and is centrally coordinated by NIHR, Evaluation, Trials and Studies Coordinating Centre (NETSCC). It brings patients, carers and clinicians together in PSPs to identify and prioritise the unanswered questions about the effects of treatments that they agree are most important [7]. While the JLA PSP framework is a tried and tested methodology for treatment uncertainties, this is the first PSP concerned with research methodology uncertainties. Therefore, the methods used during the study were developed to accommodate a focus on methodological rather than treatment uncertainties while utilising the JLA PSP framework.

The purpose of the study was to identify unanswered questions around trial recruitment research, and then prioritise these based on agreement from across the relevant trial stakeholder groups.

## Methods

The PRioRiTy PSP was formally initiated in a meeting at the International Clinical Trials Methodology Conference in November 2015, where members (from the HRB-TMRN, Trial Forge (http://trialforge.org), NIHR Evaluation, Trials and Studies Coordinating Centre (NETSCC; http://www.southampton.ac.uk/netscc/index.page) and the MRC-HTMR (https://www.methodologyhubs.mrc.ac.uk)) agreed the scope and nature of the project. Methodological uncertainties around trial recruitment were defined as the recruitment challenges encountered by recruiters, trial designers and persons being recruited to randomised trials of health care interventions. To collect a representative range of opinions, the PRioRiTy working group brought people together from across the UK and Ireland who were, or had been, involved directly in any aspect of randomised trials.

### Establishing the Steering Group

A Steering Group to oversee the PSP was established in accordance with JLA guidance and held its first meeting in May 2016. Potential steering group members were identified by the HRB-TMRN and the JLA through an open and inclusive approach of peer knowledge and consultation with respective networks and existing contacts. Membership included equal representation from researchers, clinicians, trial experts, the public and/or their representatives, as well as JLA staff. The primary role of the Steering Group was to discuss and agree the strategic orientation and processes of the PRioRiTy PSP. The protocol was developed in collaboration with the JLA and with reference to the JLA guidebook modified as appropriate for the methodology, rather than treatment, focus of the PSP.

### Identifying and inviting potential partners

The first stage in the process was to invite potential partner organisations to engage with the PRioRiTy PSP. As per the JLA PSP guidance, industry representation was excluded from this PSP, as traditionally the health research agenda has been largely directed by the industry agenda, with the voice of patients and carers rarely included. However, the unique subject of the PRioRiTy PSP meant that some changes to the usual exclusion criteria of stakeholder groups were made. The JLA PSP process does not usually invite representatives of the research community (e.g. front line research staff and methodologists) who are not also clinicians, patients or carers to participate in the priority setting process; this stakeholder group was included in this PSP given their various roles in randomised trials.

Potential partner organisations were identified through a process of peer knowledge and consultation, through the Steering Group members’ respective networks and through the JLA’s existing contacts. The PRioRiTy PSP ensured that a wide range of partners representing the broad stakeholder groups across Ireland and the UK were secured so that the uncertainties gathered would be from as wide a range of potential contributors as possible. Potential partners were contacted and informed of the establishment and aims of the PRioRiTy PSP and invited to participate in the PSP. Partners were expected to help promote the PSP to their members and to encourage participation in the survey used to gather uncertainties. In line with JLA guidance, we did not include representatives of the pharmaceutical industry.

### Identifying and engaging the stakeholders

The stakeholders for this PSP (listed below) were the people to whom the online survey for the initial gathering of information around possible uncertainties was distributed:

- Members of the public who had been invited to participate in a randomised trial or participated in Trial Steering Committees (TSCs). They could be an individual or representing a patient organisation;
- Front line clinical and research staff who were or had been involved in recruitment to randomised trials (e.g. postdoctoral researchers, clinicians, nurses, midwives, allied health professionals);
- People who had established expertise in designing, conducting, analysing and reporting randomised trials (e.g. Principal Investigators/Chief Investigators);
- People who are familiar with the trial methodology research landscape (e.g. funders, programme managers, network coordinators).

### Initial survey development and deployment

Initial feedback from stakeholders on the challenges in trial recruitment that they perceived as important and wished to see addressed was sought in an online survey, which was available between July and August 2016 (4 weeks). This survey contained five questions (Table 1) asking people to consider their own experiences of being involved in randomised trials across specific areas. The specific areas or domains (planning and design, conduct, information, trial recruiters and motivation) were derived from the Online Resource for Recruitment research in Clinical triAls (ORRCA) project recruitment research domains [10]. The ORRCA project has a web-enabled database (http://www.orrca.org.uk/) populated with published and ongoing recruitment research. Respondents were invited to submit comments in an open-ended format to a broad question about each of the ORRCA domains. For example; what questions or comments do you have (if any) about improving how trials are planned and designed? They were also offered a sixth open-ended comment box for any other items not considered in the five thematic questions. The survey also asked demographic questions and asked respondents to identify which stakeholder group they belonged to.

**Table 1:** Initial PRioRiTy survey questions

1	What questions or comments do you have (if any) about improving how trials are planned and designed?
2	What questions or comments do you have (if any) about improving how trials are carried out?
3	What questions or comments do you have (if any) about the information people are given when they join a trial?
4	What questions or comments do you have (if any) about trial recruiters who invite people to take part?
5	What questions or comments do you have (if any) about the motivation of people in joining a trial?
6	Do you have any other questions or comments?

#### Collating and analysing initial survey responses and developing questions

The constant comparative method of analysis was used to identify emergent themes in the survey responses. The constant comparative analysis method is an iterative and inductive process of reducing the data through constant recoding [11]. The information obtained from the initial survey was assembled and categorised using Microsoft Excel and merged into a single database. Responses to each survey question were re-written as research questions by two members of the project team who independently extracted the data. Longer responses were broken down into several key excerpts as appropriate and multiple questions were created. Responses judged to be not relevant to trial recruitment were classed as “out of scope” and saved for future analysis. Questions with common themes and issues were merged into broader questions where appropriate and duplicates removed. The reliability of emerging themes and issues was reviewed regularly by the two project team members swapping a portion of their respective data and comparing findings for consistency. Any discrepancies or issues arising from specific responses were adjudicated by a third team member and discussed by the research team if necessary. The recruitment research domains used for the searchable database ORRCA (http://www.orrca.org.uk/)), were used as a framework to aid this process [10]. This allowed members of the team to use the same classification of research questions across recruitment themes. A literature review was conducted in parallel with this process to ensure that all of the included questions in the interim survey were questions for which there was insufficient evidence to adequately consider them as being unanswered. Our search strategy was developed by reviewing and combining search strategies from the Cochrane systematic review on “Strategies to improve recruitment to randomised controlled trials” [4] and from the ORRCA project [10]. The search was run (July 2016) across MEDLINE, EMBASE, Cochrane Database of Systematic Reviews, Social Sciences Citation Index and ERIC. The findings of relevant, up to date (published in the proceeding 3 years as per standard JLA guidance) systematic reviews identified were mapped to the questions and reviewed by at least two members of the Steering Group (see Appendix).

#### Development of the interim survey

A second follow-up interim survey was conducted to prioritise and shortlist the newly developed questions. The interim survey was open during November 2016 (3 weeks) and asked the stakeholders to select up to 10 important questions. Invitations to this survey were not restricted to those participating in the initial survey. The resulting shortlist of questions for research was cross referenced with the identified systematic reviews. Questions were confirmed as unanswered if there was no systematic review of research evidence addressing the recruitment question or if a systematic review of research evidence confirmed that the recruitment question remains unanswered. The literature review to establish if the proposed research questions were unanswered confirmed that all 31 questions formulated from the initial survey where included in the interim survey.

#### Survey dissemination and promotion

While no formal target sample size was set for both surveys, the number of surveys being returned by each stakeholder group was monitored on a weekly basis. Where any stakeholder group participation in the survey was < 10% of the total responders, efforts were made to ensure targeted promotion of the survey among that group across the relevant partners.

Both the initial survey and the interim survey to collect recruitment uncertainties were constructed in SurveyMonkey® and embedded into the PRioRiTy sub-page of the HRB-TMRN website. The survey link was distributed by email and the survey was also available in paper format if participants preferred this format. Steering Group members and partners were asked to promote the PSP and surveys to stakeholders via email, web sites, relevant meetings, social media and any other opportunities that arose. A social media promotion plan was developed, with all Steering Group members requested to use pre-worded tweets, which included the link to the survey. No incentives were offered for return of the survey.

### Voting and ranking survey items

The Steering Group used ranked weighted scores to decide which of the interim survey research questions to take forward to the final priority setting workshop. We followed the standard JLA approach as described in detail in the JLA Guidebook (www.jla.nihr.ac.uk/jla-guidebook/). Response counts were allocated for each research question across each stakeholder group. Summed scores from each stakeholder group were calculated separately. For each of the stakeholder groups, the highest ranked question was allocated a maximum score (for example if there were 30 questions in the list, the number 1 ranked question would be assigned a score of 30), the next one down a lower score (i.e. minus 1) and so on down the list, until the lowest ranked question received the lowest score (i.e. 1). To obtain the overall ranking, the scores for each question from each of the stakeholder groups were added together. The question with the highest aggregated score was ranked number 1 overall and the lowest score was ranked lowest overall. This gave the overall interim ranking to the research questions and the rankings for each of the stakeholder groups, whilst minimising bias owing to numbers of responses from each stakeholder group.

### Priority setting workshop

A final prioritisation workshop was held in December 2016 in Birmingham, UK, in order to condense the number of questions generated by the stakeholder surveys to (a minimum of) a “Top 10” list of research questions agreed by consensus. The workshop followed the standard JLA approach as described in detail in the JLA Guidebook (www.jla.nihr.ac.uk/jla-guidebook/) and was facilitated by the JLA’s Senior Advisor. Reimbursement of expenses (all members) and remuneration for people’s time (members of the public only) was guided by the INVOLVE UK guidelines. All expenses were processed centrally by the JLA for all stakeholders. Streamlining of this process through a single line of communication facilitated appropriate procurement, efficient submission of expenses and timely reimbursement.

## Results

### Initial survey

#### Completeness of initial survey

The initial survey was completed by 790 respondents with 382 (48%) of those answering at least one of the open ended questions. Only one person requested a hard copy of the initial survey. Completeness of the initial survey across survey sections is outlined in Table 2.

**Table 2:** Completeness of initial survey

Demographic questions	Number	Completed (%)
Consent to participate (yes)	790	100%
Age (scale)	777	98%
Respondent’s role in trials	717	91%
Where respondent lives	720	91%
Gender	711	90%
Affiliated trial subject	711	90%
Wish to be involved in future research (yes/no)	713	90%
Specific open-ended feedback questions		
“How trials are planned and designed”	382	48%
“How trials are carried out”	350	44%
“Information people are given when they join a trial”	359	45%
“Trial recruiters who invite people to take part”	291	37%
“The motivation of people in joining a trial”	314	40%
“Other questions or comments”	149	19%

#### Demographic information – initial survey

The proportion of completed surveys from each stakeholder group is presented in Table 3. The stakeholder group with the highest response were researchers involved in recruiting participants (21%, *n* = 150). The number of completed survey responses across stakeholder groups, ranged from 61 to 150 (see Table 3).

**Table 3:** Initial survey respondent roles

Which one of the following best describes your main role in a randomised trial?		
Answer options	Number	Percentage
A person invited to take part in a trial	83	12
A researcher involved in recruiting participants	154	21
A non-researcher (e.g. clinician or health professional) involved in recruiting participants	77	11
A principal/chief investigator	124	17
A researcher involved in aspects of the trial other than frontline recruitment	183	26
A trial methodologist (someone who specializes in the methods of how trials are designed, run, analysed and reported)	87	12
Other (please specify)	9	1
Total^a^	717	100

^a^Data were missing in 73 respondents

Information about the clinical subject area of experience was available for 711 respondents. The highest proportion of respondents identified cancer (18%), followed by neurology – neurodegenerative diseases, vascular diseases (12%) (Table 4).

**Table 4:** Summary of initial survey respondents affiliated trial subject areas

Trial subject area	Number	Percentage
Oncology/haematology – cancer	142	20
Neurology – neurodegenerative diseases, vascular Diseases	93	13
Cardiovascular diseases	44	6
Mental health	42	6
Metabolism – diabetes mellitus	38	5
Orthopaedics/musculoskeletal	34	5
Reproductive Health	34	5
Gastroenterology – hepatology, nephrology	29	4
Paediatrics/neonates	22	3
Respiratory	20	3
Inflammatory conditions (e.g. osteoarthritis, rheumatoid arthritis, fibromyalgia)	15	2
Surgery	15	2
Ophthalmology	14	2
Vaccines – preventive vaccines	13	2
Dementia/ageing	12	2
Infectious diseases	9	1
Dermatology	7	1
Palliative care	4	1
Involved in multiple trials	38	5
Other	79	11
Total^a^	704	100%

^a^Data were missing or response was “Do not know” in 86 respondents

Respondents to the survey were predominantly from England (74%), followed by the Republic of Ireland (17%), Scotland (4%), Wales (2%) and Northern Ireland (< 1%).

### Initial survey responses, collating themes and merging questions

A total of 1880 questions for research were formulated from the 790 survey responses, which had 1693 open text responses. Merging similar themed questions and removing duplicates reduced this to 496 questions. Where a duplicate question was removed, the total cumulative frequency of the number of times this question was mentioned across stakeholders was noted and each new version of the database was saved to allow traceability of concepts and questions from verbatim quotations from individual respondents.

Where appropriate, questions were merged into broader questions following review and discussion with the steering group. Finally, questions asked by more than 15 people and/or at least 6 of the 7 stakeholder groups were selected to progress to the interim survey of stakeholders. These criteria were developed through consultation with the Steering Group after presenting data across stakeholder groups. This resulted in the inclusion of 31 unique questions for research.

### Interim survey

#### Completeness of interim survey

The interim survey was completed by 815 respondents (female 71%, male 29%), of whom 100% selected at least one research question for prioritisation.

#### Demographic information – interim survey

A total of 802 respondents provided information on their stakeholder group. A full breakdown of affiliated roles is presented in Table 5.

**Table 5:** Interim survey respondent roles

Which one of the following best describes your main role in a randomised trial?		
Answer options	Number	Percentage
A person invited to take part in a trial	108	13
A researcher involved in recruiting participants	146	18
A non-researcher (e.g. clinician or health professional) involved in recruiting participants	63	8
A principal / chief investigator	186	23
A researcher involved in aspects of the trial other than frontline recruitment	206	25
A trial methodologist (someone who specialises in the methods of how trials are designed, run, analysed and reported)	90	11
Other	3	< 1
Total	802	98

Respondents to the interim survey were predominantly from England (77%, *n* = 603), followed by the Republic of Ireland (9%, *n* = 72), Scotland (7%, *n* = 54), Wales (5%, *n* = 38) and Northern Ireland (2%, *n* = 14).

Information about the clinical subject area of experience was available for 775 respondents (Table 6).

**Table 6:** Summary of interim survey respondents affiliated trial subject areas

Trial subject area	Number	Percentage
Oncology/haematology – cancer	161	21
Neurology – neurodegenerative diseases, vascular diseases	77	10
Cardiology – cardiovascular disease	62	8
Orthopaedics/musculoskeletal	56	7
Mental health	51	7
Metabolism – diabetes mellitus	47	6
Vaccines – preventive vaccines	45	6
Reproductive health	33	4
Critical care	24	3
Infectious diseases	21	3
Gastroenterology – hepatology, nephrology	18	2
Surgery	16	2
Respiratory	15	2
Paediatrics/neonates	13	2
Inflammatory conditions (e.g. osteoarthritis, rheumatoid arthritis, fibromyalgia)	11	1
Ophthalmology	9	1
Involved in multiple trials	34	4
Other	82	11
Total^a^	785	96

^a^Data missing in 30 respondents

### Interim survey ranking

The Steering Group followed the standard JLA approach and used ranked weighted scores across all stakeholder groups to decide which of the interim survey research questions to take forward to the final priority setting workshop. For this workshop, based on JLA experience in prioritising questions, 25 questions were brought forward for discussion and final prioritising by the group.

### Final prioritization workshop

The final prioritisation workshop took place in Birmingham in December 2016 and a final Steering Group meeting was held the following day to review the results of the workshop. There were 31 participants representing the stakeholder groups who were invited to the face-to-face final priority setting workshop and on the day 26 participants attended. This was made up of 10 public members from trials or trial steering committees, 7 frontline researchers or non-researchers involved in recruitment, 6 trial methodologists, and 3 researchers or principal investigators. Some members of the PRioRiTy Steering Group attended as observers. The 26 participants were divided into three groups with a JLA facilitator for each group. Each group was provided with the shortlisted questions in individual question cards with stakeholder group rankings from the voting process and an example quote from the original survey submissions noted on the back of them. The questions had been sent to the participants prior to the meeting so that they could have some time to familiarise themselves with the list and decide on what was important to them. The facilitators then guided the participants through the process of discussing the questions and agreeing, by consensus, a “Top 10” from within them.

The group agreed that a ranked “Top 10” list would be created and ranking for an additional ten questions (11–20) would also be carried out. Therefore, the PRioRiTy list of research questions features a list of both the “Top 10” (Table 7) and the research questions ranked 11–20 are available (www.priorityresearch.ie).

**Table 7:** The “Top 10” research questions prioritised

Overall ranking	Uncertainty as research question
1	How can randomised trials become part of routine care and best utilise current clinical care pathways?
2	What information should trialists communicate to members of the public who are being invited to take part in a randomised trial in order to improve recruitment to the trial?
3	Does patient/public involvement in planning a randomised trial improve recruitment?
4	What are the best approaches for designing and delivering information to members of the public who are invited to take part in a randomised trial?
5	What are the barriers and enablers for clinicians/healthcare professionals in helping conduct randomised trials?
6	What are the key motivators influencing members of the public’s decisions to take part in a randomised trial?
7	What are the best approaches to ensure inclusion and participation of under-represented or vulnerable groups in randomised trials?
8	What are the best ways to predict recruitment rates to a randomised trial and what impact do such predictions have on recruitment?
9	What are the best approaches to optimise the informed consent process when recruiting participants to randomised trials?
10	What are the advantages and disadvantages to using technology during the recruitment process?

**Fig. 1:**
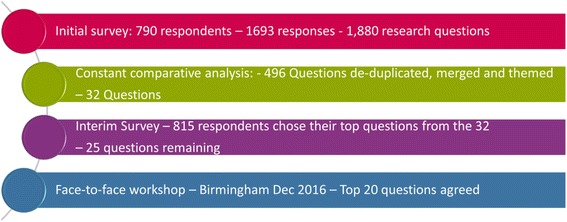
Collating and analysing survey responses and developing questions for consensus

The process of managing the data analysis is illustrated in Fig. 1.

### Accessibility of the research question list and repository of relevant research

The top 10 questions for research can be viewed on a user-friendly, dedicated website (www.priorityresearch.ie). Research teams conducting specific research relevant to any of the PRioRiTy research questions are requested to submit basic details of their work to the HRB-TMRN through the PRioRiTy website as a repository and central record platform for the level of research ongoing for each research question. This resource will be maintained by the HRB-TMRN and will be freely searchable and accessible.

## Discussion

The JLA process of priority setting through partnership and consensus is already well-established around treatment uncertainties. We believe this is the first time the process has been used to identify uncertainties around research methodology.

This Priority PSP identified that stakeholders believe that to improve the process of how people are recruited to randomised trials, research attention needs to focus on normalising trials as part of clinical care, enhancing communication, addressing barriers, enablers and motivators around participation and exploring greater public involvement in the research process.

### Challenges encountered

While using a modified JLA process contributed greater efficiency to the study, there were several challenges throughout the process that had to be addressed at each stage. Given that the topic under discussion was research methodology, we were concerned that the voice of the public may have been overshadowed by that of research academics and practitioners. This issue was discussed at all stages, from completing the surveys through to the face-to-face workshop. To minimise this risk, regular checking of survey responses and dedicated promotion of the survey to the public, in particular, was carried out. Our experience suggests that engaging people to take part in research about how to improve recruitment of people into trials is difficult and this is reflected in a lower response to the initial and interim surveys from members of the public relative to other stakeholder groups. However, our efforts from the beginning of the work, and in particular our Steering Group public representatives, helped ensure that the involvement of the public was meaningful and relevant throughout the project, that the process and language was accessible and that public representatives understood the work and felt that they could contribute on an equal basis.

The face-to-face workshop was not without its challenges. The workshop participants were assigned to small groups initially, each of which had a mixture of stakeholders. The dynamic of each group was slightly different, due to different backgrounds, perspectives, personalities, communication styles, expertise and levels of confidence. The groups were required to cover some complex issues regarding research methodology in discussing the uncertainties presented and reach a consensus around prioritising them. The discussions were robust and lively with each person, as might be expected, taking strong ownership of their own priorities and personal preferences. The presence of an experienced facilitator to moderate those discussions was fundamental in ensuring full, fair, respectful and equal participation. The facilitators took steps to ensure that no one dominated, or was excluded from, the discussion. Pragmatism was required within each group to reach acceptable compromises and revision of opinions in the search for consensus. The small groups then reconvened to one large group to agree the final “top ten”. Good facilitation was again instrumental here to reach a democratic compromise where no-one felt coerced to let go of the priorities around which they felt strong ownership.

Another significant challenge centred on the language used during all stages of this PSP, for example, how the term “trial participants” was interpreted; some viewed this as patients only, others thought of clinicians and healthcare workers as “trial participants”. Other items centred around classifying “hard-to-reach” or “vulnerable” groups, for which public and patient contributors on the Steering Group were able to provide more appropriate wording such as “seldom-heard” groups. To overcome issues around appropriate language and in a bid to provide distilled language, the “GET IT” glossary (http://getitglossary.org/ [12] was used and words were hyperlinked to their specific translation on all materials, from website to initial survey to interim survey.

The submission of a large volume of data in the initial survey raised challenges around data management. The large volume of data needed to be interpreted, categorised and combined into themes whilst at the same time remaining true to the richness of the submission. The two members of the project group independently analysing the data, randomly and regularly checked a sample of each other’s interpretation and categorisation with a third member adjudicating on any disagreements. All data were filed in a manner that facilitates tracking each recruitment question back to the original submission by the stakeholder.

The involvement of a wide stakeholder group in a face-to-face meeting also presented new challenges to the team, for which the JLA have considerable experience. For example, while the research team may be very familiar with using email for communication, this was less so for some of the invited participants, with many not familiar with using email attachments or checking their email infrequently. The team adapted by communicating via phone and text, making sure that every email was accompanied by a phone reminder or text message where needed.

Many of the challenges we encountered have previously been described by others attempting public involvement with research [13–15]. Similar to their experiences, we found that a flexible and responsive approach was needed to successfully address the challenges.

#### Implications of PRioRiTy

We believe that the PSP model was applied successfully to the identification and prioritisation of methodological uncertainties in trial recruitment and that such an approach has merit in identifying uncertainties in other trial processes e.g. retention, reporting etc. The use of the PSP process for this project also provided some very useful learning around the successful engagement of the public and patients in the conversation about trial methodology. Given that the challenge of recruiting to trials is an international problem, repeating this priority setting exercise in other countries may need to be considered. The PRioRiTy study was Ireland and UK centred but having found the PSP process to be effective it could be replicated in other jurisdictions where different trial infrastructural supports are available. International collaborations should now be fostered and the efforts of research groups all over the globe combined to address these prioritised methodological questions.

## Conclusion

This bringing together of people, engaged researchers, clinicians and the public in an exercise of discussion, knowledge exchange and consensus, in identifying, agreeing, prioritising and disseminating a list of the most important methodological uncertainties surrounding recruitment to trials. Methodology research, such as the PRioRiTy PSP, is an essential adjunct to clinical research. Such so called “research on research” is acknowledged as an important contributor to reducing waste and inefficiencies in research [16]. The critical end point of the PRioRiTy PSP is a top ten list of trial recruitment uncertainties, determined by those directly involved in trials, which will inform future research around developing more effective recruitment procedures and processes that encourage participation in randomised trials. The investigation of, and answers to, the research questions identified by PRioRiTy will inform future research designs and increase the efficiency of recruitment to trials. This, according to Salman et al. (2014), will minimise avoidable sources of waste and inefficiency in research [17]. Further, ongoing partner engagement in designing future studies around the identified priorities will be encouraged and supported so that trials might be better designed and implemented in the future. Recruitment to trials is an international issue that would be best addressed by engaging in international collaborations for those ongoing partner engagements.

International research groups are encouraged to collaborate and contribute evidence to answer the prioritised recruitment questions. Researchers are encouraged to identify opportunities for building robust proposals to answer these priorities and research funders are encouraged to integrate the priorities into their organisational plans, research strategies and funding calls.

## Appendix

**Table 8:** Evidence checking

Uncertainty as research question	Evidence reviewed (878 screened, 76 full text, 34 extracted)
1. How can randomised trials become part of routine care and best utilise current clinical care pathways?	• No evidence available
2. What information should trialists communicate to members of the public who are being invited to take part in a randomised trial in order to improve recruitment to the trial?	• No evidence available
3. Does patient/public involvement in planning a randomised trial improve recruitment?	• No evidence available
4. What are the best approaches for designing and delivering information to members of the public who are invited to take part in a randomised trial?	• Bonevski, B.; Randell, M.; Paul, C.; Chapman, K.; Twyman, L.; Bryant, J.; Brozek, I.; Hughes, C. Reaching the hard-to-reach: a systematic review of strategies for improving health and medical research with socially disadvantaged groups. BMC Medical Research Methodology 2014; 14:42. • Synnot, A.; Ryan, R.; Prictor, M.; Fetherstonhaugh, D.; Parker, B.Audio-visual presentation of information for informed consent for participation in clinical trials. Cochrane Database of Systematic Reviews 2014;9(5):CD003717. • Treweek, S.; Lockhart, P.; Pitkethly, M.; Cook, J. A.; Kjeldstrom, M.; Johansen, M.; Taskila, T. K.; Sullivan, F. M.; Wilson, S.; Jackson, C.; Jones, R.; Mitchell, E. D. Methods to improve recruitment to randomised controlled trials: Cochrane systematic review and meta-analysis. BMJ Open 2013;3(2):
5. What are the barriers and enablers for clinicians/healthcare professionals in helping conduct randomised trials?	• Newington, L.; Metcalfe, A. Researchers’ and clinicians’ perceptions of recruiting participants to clinical research: a thematic meta-synthesis. Journal of Clinical Medicine Research 2014;6(3):162-72
6. What are the key motivators influencing members of the public’s decisions to take part in a randomised trial?	• Limkakeng Limkakeng, A.; Phadtare, A.; Shah, J.; Vaghasia, M.; Wei, D. Y.; Shah, A.; Pietrobon, R.Willingness to participate in clinical trials among patients of Chinese heritage: a meta-synthesis. PLoS ONE [Electronic Resource] 2013; 8(1):e51328. • Nalubega, S.; Evans, C. Participant views and experiences of participating in HIV research in sub-Saharan Africa: a qualitative systematic review. JBI Database Of Systematic Reviews And Implementation Reports 2015;13(5):330-420 • Rivers, D.; August, E. M.; Sehovic, I.; Lee Green, B.; Quinn, G. P. A systematic review of the factors influencing African Americans’ participation in cancer clinical trials. Contemporary Clinical Trials 2013;35(2):13-32 • Wilman, E.; Megone, C.; Oliver, S.; Duley, L.; Gyte, G.; Wright, J. M.The ethical issues regarding consent to clinical trials with pre-term or sick neonates: a systematic review (framework synthesis) of the empirical research.Trials [Electronic Resource] 2015;16:502
7. What are the best approaches to ensure inclusion and participation of under-represented or vulnerable groups in randomised trials?	• Bellera, C.; Praud, D.; Petit-Moneger, A.; McKelvie-Sebileau, P.; Soubeyran, P.; Mathoulin-Pelissier, S. Barriers to inclusion of older adults in randomised controlled clinical trials on non-Hodgkin’s lymphoma: a systematic review. Cancer Treatment Reviews 2013;39(7):812-817 • Cooper, C.; Ketley, D.; Livingston, G. Systematic review and meta-analysis to estimate potential recruitment to dementia intervention studies.International Journal of Geriatric Psychiatry 2014;29(5):515-25 • Nicholson, L. M.; Schwirian, P. M.; Groner, J. A. Recruitment and retention strategies in clinical studies with low-income and minority populations: progress from 2004 to 2014. Contemporary Clinical Trials 2015;45(Pt A):34-40
8. What are the best ways to predict recruitment rates to a randomised trial and what impact do such predictions have on recruitment?	• Cooper, C. L.; Hind, D.; Duncan, R.; Walters, S.; Lartey, A.; Lee, E.; Bradburn, M.A rapid review indicated higher recruitment rates in treatment trials than in prevention trials. Journal of Clinical Epidemiology 2015;68(3):347-54
9. What are the best approaches to optimise the informed consent process when recruiting participants to randomised trials?	• Farrell, E. H.; Phillips, K.; Morgan, B.; Savage, K.; Lewis, V.; Whistance, R. N.; Kelly, M.; Mann, M.; Blazeby, J. M.; Kinnersley, K.; Edwards, A. G. K. Audio-visual aids for informed consent for invasive healthcare procedures: A systematic review. British Journal of Surgery 2013;100:60 • Gillies, K.; Cotton, S. C.; Brehaut, J. C.; Politi, M. C.; Skea, Z. Decision aids for people considering taking part in clinical trials. Cochrane Database of Systematic Reviews 2015;11:CD009736 • Halkoaho, A.; Pietila, A. M.; Ebbesen, M.; Karki, S.; Kangasniemi, M. Cultural aspects related to informed consent in health research: A systematic review. Nursing Ethics 2015;23(6):698-712 • Eltorki, M.; Uleryk, E.; Freedman, S. B. Waiver of informed consent in pediatric resuscitation research: a systematic review. Academic Emergency Medicine 2013;20(8):822-34 • Boland, J.; Currow, D. C.; Wilcock, A.; Tieman, J.; Hussain, J. A.; Pitsillides, C.; Abernethy, A. P.; Johnson, M. J. A systematic review of strategies used to increase recruitment of people with cancer or organ failure into clinical trials: implications for palliative care research. Journal of Pain & Symptom Management 2015;49(4):762-772.e5 • Montalvo, W.; Larson, E. Participant comprehension of research for which they volunteer: a systematic review. Journal of Nursing Scholarship 2014;46(6):423-31 • Nishimura, A.; Carey, J.; Erwin, P. J.; Tilburt, J. C.; Murad, M. H.; McCormick, J. B. Improving understanding in the research informed consent process: a systematic review of 54 interventions tested in randomized control trials. BMC Medical Ethics 2013;14:28 • Treweek, S.; Lockhart, P.; Pitkethly, M.; Cook, J. A.; Kjeldstrom, M.; Johansen, M.; Taskila, T. K.; Sullivan, F. M.; Wilson, S.; Jackson, C.; Jones, R.; Mitchell, E. D. Methods to improve recruitment to randomised controlled trials: Cochrane systematic review and meta-analysis. BMJ Open 2013;3(2): • Synnot, A.; Ryan, R.; Prictor, M.; Fetherstonhaugh, D.; Parker, B. Audio-visual presentation of information for informed consent for participation in clinical trials. Cochrane Database of Systematic Reviews 2014;9(5):CD003717 • Wilman, E.; Megone, C.; Oliver, S.; Duley, L.; Gyte, G.; Wright, J. M. The ethical issues regarding consent to clinical trials with pre-term or sick neonates: a systematic review (framework synthesis) of the empirical research. Trials [Electronic Resource] 2015;16:502
10. What are the advantages and disadvantages to using technology during the recruitment process?	• No evidence available
